# Controlling the Mixing Sequence of the Reactive Compatibilizer SAN-g-Epoxy in PBT/ABS Blends: Enhancing Mechanical and Thermomechanical Performance Through Interfacial Engineering

**DOI:** 10.3390/ijms27083343

**Published:** 2026-04-08

**Authors:** Carlos Bruno Barreto Luna, Eduardo da Silva Barbosa Ferreira, Edson Antonio Dos Santos Filho, Fabiano Santana da Silva, José Vinícius Melo Barreto, Danilo Diniz Siqueira, Renate Maria Ramos Wellen, Edcleide Maria Araújo

**Affiliations:** 1 Laboratório de Processamento de Polímeros, Unidade Acadêmica de Engenharia de Materiais, Universidade Federal de Campina Grande, Campina Grande 58429-900, PB, Brazil; eduardosbf95@gmail.com (E.d.S.B.F.); edson.a.santos.f@gmail.com (E.A.D.S.F.); sunsolaris8@hotmail.com (F.S.d.S.); viniciusbarreto89@hotmail.com (J.V.M.B.); edcleidemaraujo@gmail.com (E.M.A.); 2 MackGraphe, Mackenzie Institute for Research in Graphene and Nanotechnologies, São Paulo 01302-907, SP, Brazil; danilo.siqueira@mackenzie.br; 3 Department of Materials Engineering, Federal University of Paraíba, Cidade Universitária, João Pessoa 58051-900, PB, Brazil; wellen.renate@gmail.com

**Keywords:** PBT, ABS, polymer blends, reactive compatibilization, performance

## Abstract

Polymer blends constitute a strategy for tailoring the properties of commercial polymers, leading to the development of materials designed for specific applications. In this work, the effect of the mixing sequence of the reactive compatibilizer styrene–acrylonitrile functionalized with epoxy groups (SAN-g-Epoxy) on the performance of poly(butylene terephthalate) (PBT)/acrylonitrile–butadiene–styrene (ABS) blends was investigated. PBT/ABS blends (60/40 wt%) were prepared by reactive extrusion in a twin-screw extruder followed by injection molding, incorporating five parts per hundred resin (phr) of SAN-g-Epoxy through different mixing sequences, aiming to understand how the processing order influences interfacial reactions, morphology, and the final properties of the material. The results indicated that SAN-g-Epoxy promotes reactive compatibilization between PBT and ABS, as evidenced by a significant increase in torque and complex viscosity, as well as by an increase in the intensity of the carbonyl band in the Fourier transform infrared spectroscopy (FTIR) spectra. By scanning electron microscopy (SEM), the presence of the compatibilizer resulted in a pronounced morphological refinement of the dispersed ABS phase, reducing the average particle size from approximately 4.34 µm to about 0.47–0.54 µm. Among the processing strategies, the route (PBT/SAN-g-Epoxy) + ABS exhibited the best mechanical performance under impact, reaching 206.7 J/m. However, the simultaneous mixing sequence PBT/ABS/SAN-g-Epoxy showed the best balance of properties, with gains of 203% in impact strength, 8.8% in elastic modulus, and 40.1% in heat deflection temperature (HDT) compared to neat PBT. The results indicate that PBT can be improved and tailored for engineering applications.

## 1. Introduction

Polymer blends represent an efficient technological strategy for adapting existing materials to new application requirements without the need to synthesize new polymers [1,2]. This approach enables the adjustment of properties of commercial polymers, reducing costs while improving performance [3,4]. In addition, it allows the customization of materials for specific sectors, such as packaging, transportation, and electronic devices, promoting technically viable and economically feasible solutions [5]. The development of polymer blends has significant technological relevance because it can be achieved through processing techniques already widely used in the plastics transformation industry, such as extrusion and injection molding [6,7]. This characteristic facilitates the incorporation of polymer blends into existing industrial production lines without the need for significant modifications to processing infrastructure [8,9]. As a result, it becomes possible to modify or optimize the performance of commercial polymers in an economically viable manner, enabling the large-scale production of materials with properties tailored to the specific requirements of different industrial applications [10,11]. In this context, polymer blends have been widely explored as an approach for the toughening of polymers [12,13]. However, the main challenge lies in properly controlling the formulation in order to achieve a balance between mechanical and thermomechanical properties.

Poly(butylene terephthalate) (PBT) is an engineering polyester widely used due to its good dimensional stability, chemical resistance, processability, and good mechanical properties under tensile loading [14]. However, some limitations restrict its performance in certain applications, such as low impact strength and relative sensitivity to notch-induced fracture [15]. In view of these limitations of PBT, modification of its properties becomes necessary to expand its range of applications. In this context, the development of polymer blends with a PBT matrix emerges as a strategy to tailor mechanical and thermomechanical characteristics, thereby increasing its potential to more effectively meet technological and industrial demands [16,17,18]. There are scientific reports on the development of PBT blends with investigations focusing on toughening [19,20], morphological evolution [21,22], rheological behavior [23,24], crystallization [25,26], and thermal properties [27,28], demonstrating the effectiveness of this approach for tailoring the properties of PBT.

Among the commercially available impact modifiers with wide availability, ABS (acrylonitrile–butadiene–styrene) stands out due to its good mechanical properties, excellent processability, and high capacity for energy dissipation under impact, creating potential for the production of PBT/ABS blends [29]. However, to produce high-performance PBT/ABS blends, it becomes essential to carry out a compatibilization process, which expands the technological potential of these blends for engineering applications [30]. The direct blending of PBT/ABS presents limited thermodynamic affinity and depends on the acrylonitrile content, which requires the addition of a compatibilizing agent [31]. Without this compatibilization strategy, PBT/ABS blends tend to exhibit phase separation and low interfacial adhesion, which compromises stress transfer efficiency and mechanical performance [32].

The industrial key to optimizing the performance of PBT/ABS blends lies in the appropriate selection of the compatibilizing agent, which requires investigations to determine the suitable amount and type of functional group, considering the potential of PBT to undergo reactive compatibilization. In this context, PBT/ABS blends have been compatibilized with styrene–acrylonitrile–glycidyl methacrylate (SAN-g-GMA) [33], ethylene–glycidyl methacrylate (E-GMA) [34], methyl methacrylate–glycidyl methacrylate–ethyl acrylate (MGE) [35], poly(ethylene-co-glycidyl methacrylate) (E-GMA) [36], methyl methacrylate–glycidyl methacrylate (MMA-GMA) [37], styrene–acrylonitrile–maleic anhydride (SAN-g-MA) [38], styrene–maleic anhydride (SMA) [39], and styrene–ethylene/butylene–styrene grafted with maleic anhydride (SEBS-g-MA) [40], indicating that these are effective compatibilizers for improving the performance of PBT. Although several reactive compatibilizers have been investigated for PBT/ABS systems, studies exploring SAN-type copolymers functionalized with epoxy groups as compatibilizing agents are still relatively scarce, particularly regarding the influence of incorporation routes and compatibilizer distribution during reactive processing. These factors can directly influence the interfacial reaction kinetics, the in situ formation of copolymers, and, consequently, the final morphology of the blend. The selection of the epoxy-functionalized SAN copolymer (SAN-g-Epoxy) as a compatibilizing agent for PBT/ABS blends is based on a scientific hypothesis grounded in the synergy between thermodynamic affinity and chemical reactivity at the molecular level, as demonstrated in the literature [29,33]. The SAN segment exhibits high affinity with the SAN phase of ABS, promoting interfacial stabilization and reducing interfacial tension. Simultaneously, the epoxy groups react with the –OH and –COOH end groups of PBT during processing, forming interactions and generating PBT-g-SAN structures in situ. In this context, investigating different addition routes of the SAN-g-Epoxy compatibilizer in PBT/ABS blends represents an important approach to understanding how the mixing history affects the relationships between morphology and properties. Therefore, this work contributes to expanding knowledge in the field of reactive polymer blends, providing insights for the development of materials with performance tailored for engineering applications.

Therefore, this study proposes to systematically investigate different distribution strategies of the SAN-g-Epoxy compatibilizer in PBT/ABS blends during melt processing. The aim is to understand how the mixing protocol affects the reactions, as well as its consequences on the morphology, rheological behavior, thermal transitions, and mechanical properties of the system. From a molecular standpoint, this study focuses on polymer chemistry, as it demonstrates how SAN-g-Epoxy promotes interactions with PBT and ABS chains, leading to the in situ formation of grafted structures and improved molecular-level interactions between the phases.

## 2. Results

### 2.1. Torque Rheometry

**Figure 1:**
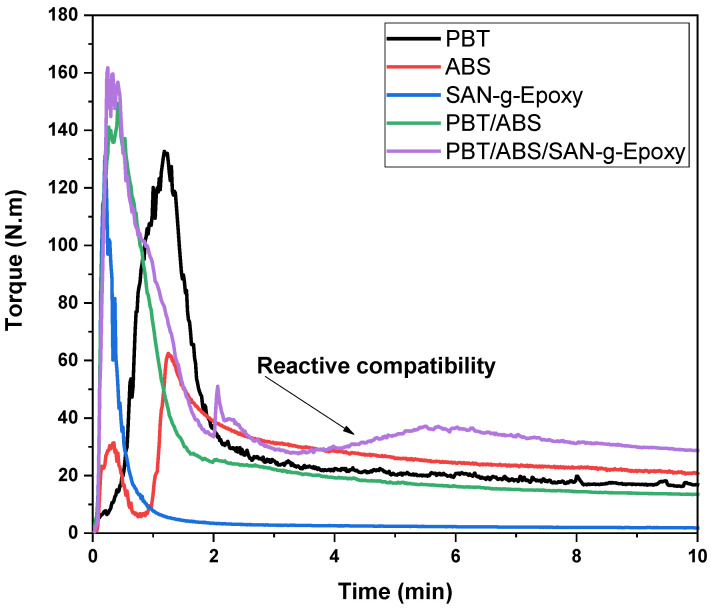
Evolution of torque rheometry for the neat polymers and the polymer blends, before and after the compatibilization process.

Torque rheometry is a sensitive analysis for monitoring variations in polymer blend formulations in the molten state, aiming to detect possible reactive events during processing. The evolution of torque over time reflects indications of structural changes associated with compatibilization reactions [41,42]. Figure 1 shows the behavior of the reactivity curves obtained by torque rheometry for the neat polymers and the polymer blends, with and without compatibilizer. The objective was to monitor whether the SAN-g-Epoxy compatibilizer was indeed capable of reacting with the PBT/ABS system. Initially, for all materials, the phenomenon of mechanical energy dissipation occurred, reaching the maximum torque. Subsequently, the materials plasticize and melt, leading to a reduction in torque until the stabilization stage is reached. In this stability plateau, viscous dissipation predominates, generating a torque response proportional to viscosity. In this case, once the stability profile of the curve is reached, the torque is considered proportional to the viscosity of the polymer system, a condition suitable for extrusion and injection molding processes [43].

**Figure 2:**
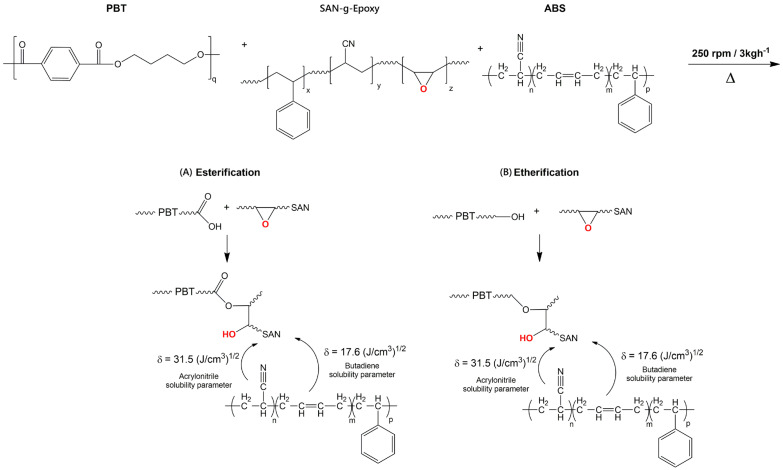
Suggested reaction mechanism for the compatibilization of the PBT/ABS/SAN-g-Epoxy blend, for the formation of: (A) ester; (B) ether. Functional group highlighted in red.

Figure 2 presents the suggested reaction mechanism for the PBT/ABS/SAN-g-Epoxy blend.

### 2.2. Oscillatory Rheology

**Figure 3:**
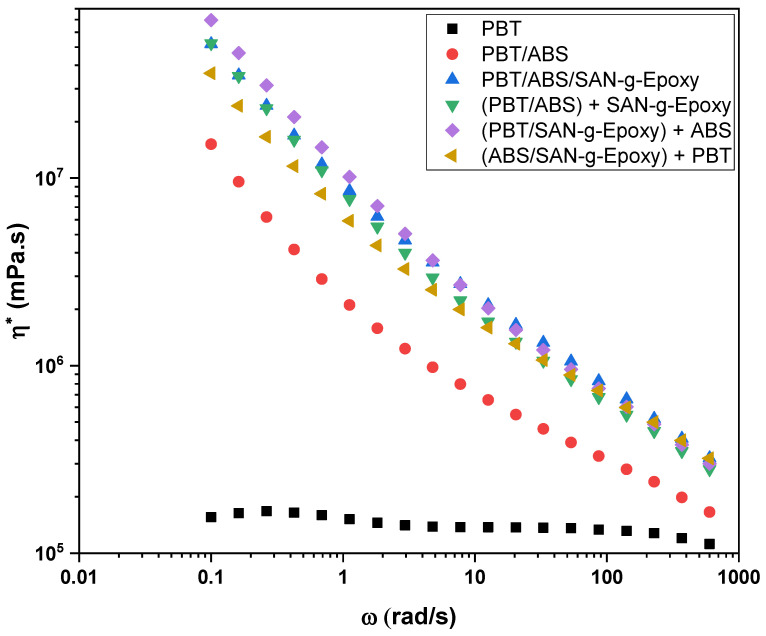
Evolution of the complex viscosity of neat PBT and the polymer blends, with and without the SAN-g-Epoxy compatibilizing agent.

Figure 3 presents the variation in complex viscosity (η*) as a function of angular frequency for neat PBT, the PBT/ABS system, and the SAN-g-Epoxy compatibilized blends obtained through different mixing protocols.

**Figure 4:**
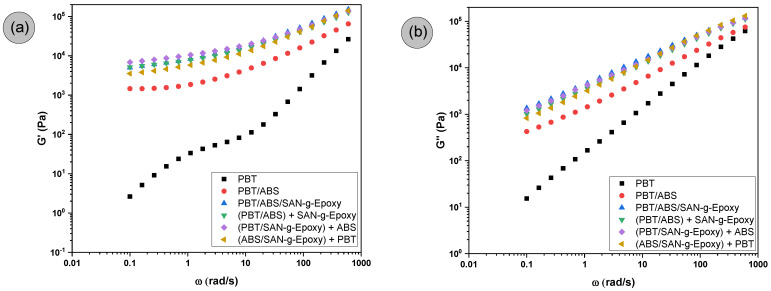
Rheological behavior of neat PBT and the PBT/ABS blends, with the compatibilizing agent and different mixing protocols, for: (**a**) Storage modulus (G′); (**b**) Loss modulus (G″).

The analysis of the viscoelastic moduli, storage modulus (G′) and loss modulus (G″), as a function of angular frequency is presented in Figure 4a,b for neat PBT, the PBT/ABS system, and the PBT/ABS/SAN-g-Epoxy blends obtained through different compatibilizer distribution routes.

### 2.3. Fourier Transform Infrared Spectroscopy (FTIR)

**Figure 5:**
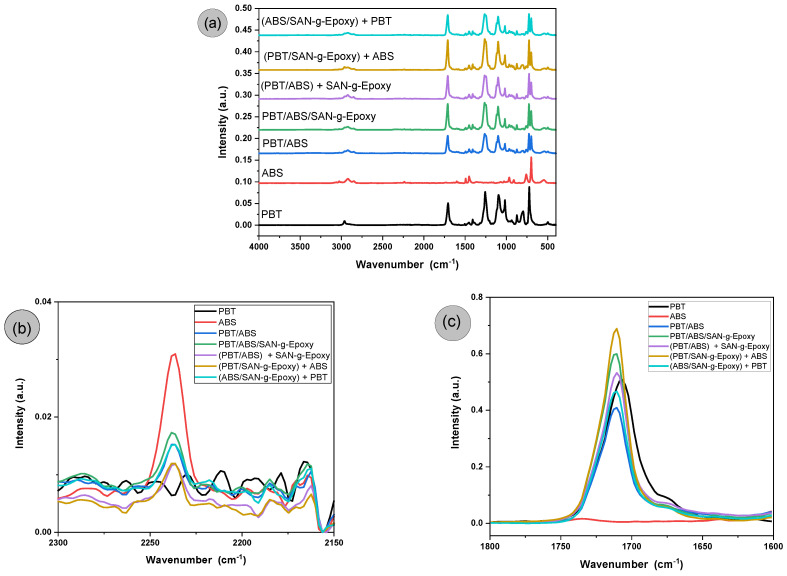
Evolution of FTIR spectra for neat PBT, ABS, and the polymer blends before and after the compatibilization process and for the different mixing protocols. (**a**) Spectral region from 4000 to 400 cm^−1^; (**b**) Magnified view of the acrylonitrile characteristic region (2235–2240 cm^−1^); (**c**) Magnified view of the carbonyl-related region (1800–1600 cm^−1^).

Figure 5a–c shows the FTIR spectra of PBT, ABS, and the polymer blends, with and without the SAN-g-Epoxy compatibilizing agent.

### 2.4. Scanning Electron Microscopy (SEM)

**Figure 6:**
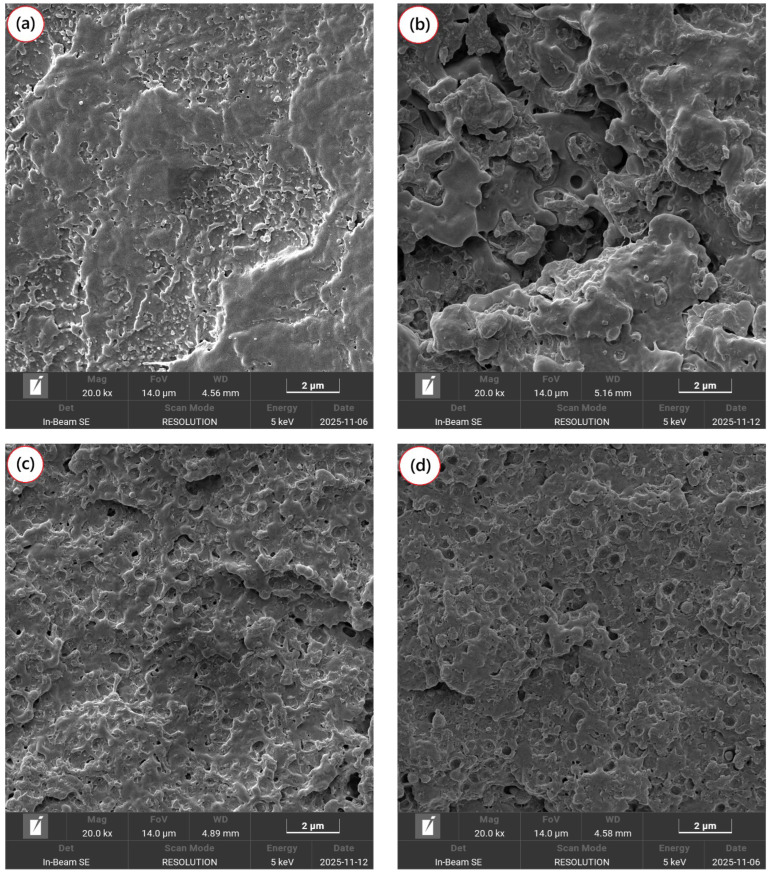

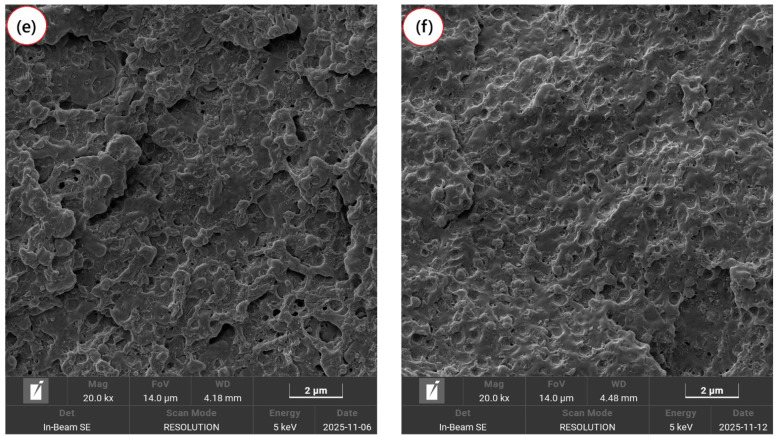
Morphological change by SEM at 20,000× magnification for: (**a**) PBT; (**b**) PBT/ABS; (**c**) PBT/ABS/SAN-g-Epoxy; (**d**) (PBT/ABS) + SAN-g-Epoxy; (**e**) (PBT/SAN-g-Epoxy) + ABS; (**f**) (ABS/SAN-g-Epoxy) + PBT.

Figure 6a–f show the fracture surfaces under Izod impact, performed at room temperature for neat PBT and the PBT/ABS blend, both without and with the addition of 5 phr of the SAN-g-Epoxy compatibilizer, as well as for the different mixing protocols.

**Figure 7:**
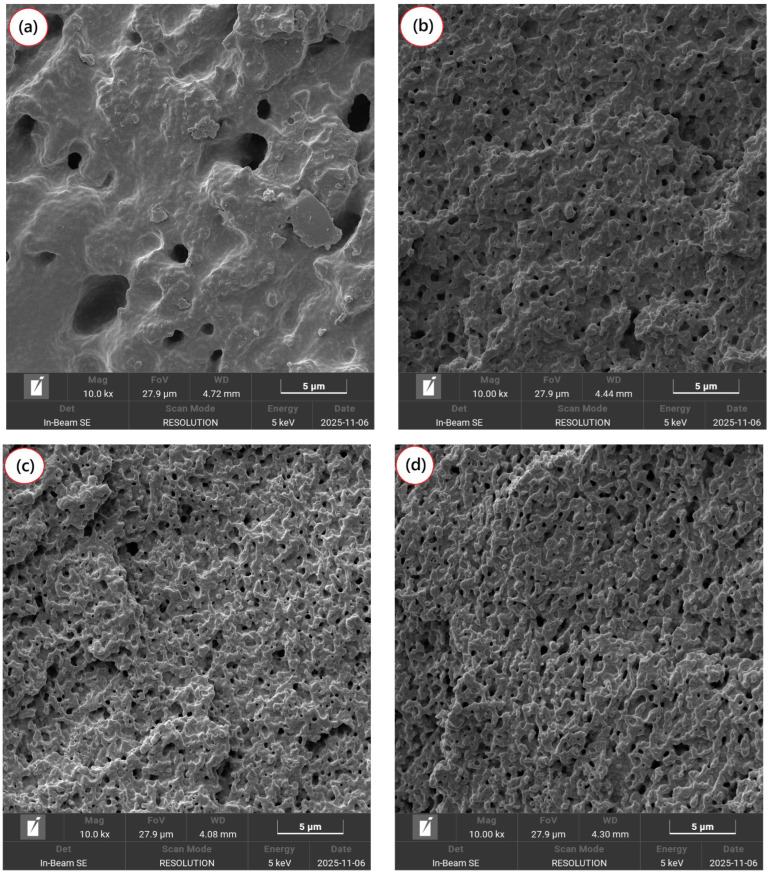

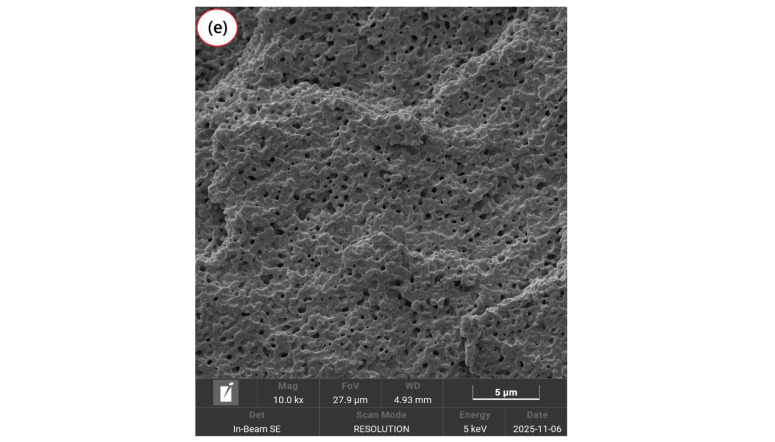
Change in morphology by SEM after selective extraction of ABS, for: (**a**) PBT/ABS; (**b**) PBT/ABS/SAN-g-Epoxy; (**c**) (PBT/ABS) + SAN-g-Epoxy; (**d**) (PBT/SAN-g-Epoxy) + ABS; (**e**) (ABS/SAN-g-Epoxy) + PBT.

Figure 7a–e shows the morphology of the PBT/ABS and PBT/ABS/SAN-g-Epoxy blends, with different compatibilizer mixing sequences, at 10,000× magnification. The selective extraction of ABS from the PBT matrix was performed using N-methyl-2-pyrrolidone (NMP) to better understand how the SAN-g-Epoxy compatibilizer modified the morphology under the different mixing sequences.

**Figure 8:**
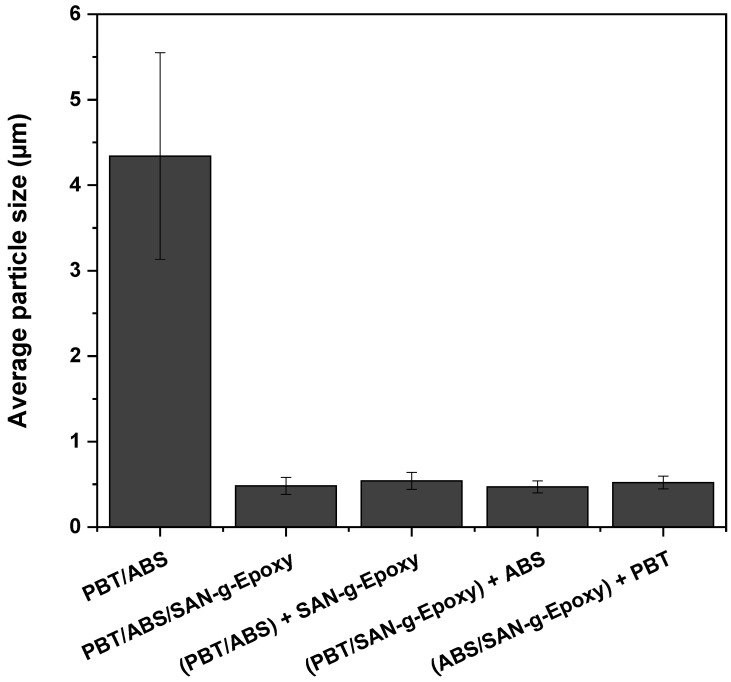
Average size of ABS particles in the polymer blends: PBT/ABS, PBT/ABS/SAN-g-Epoxy, and different mixing protocols.

Figure 8 presents the average size of ABS particles for the PBT/ABS blend, PBT/ABS/SAN-g-Epoxy, and the different mixing protocols. The particle size of the dispersed phase is critical for the toughening mechanism in polymer blends. Smaller, well-distributed domains favor energy dissipation, while larger particles reduce stress transfer efficiency, limiting improvements in impact strength [44,45]. However, the reduction in dispersed phase domain size occurs within an optimal range for each polymer matrix, which plays a significant role in the final material performance.

### 2.5. Impact Strength

**Figure 9:**
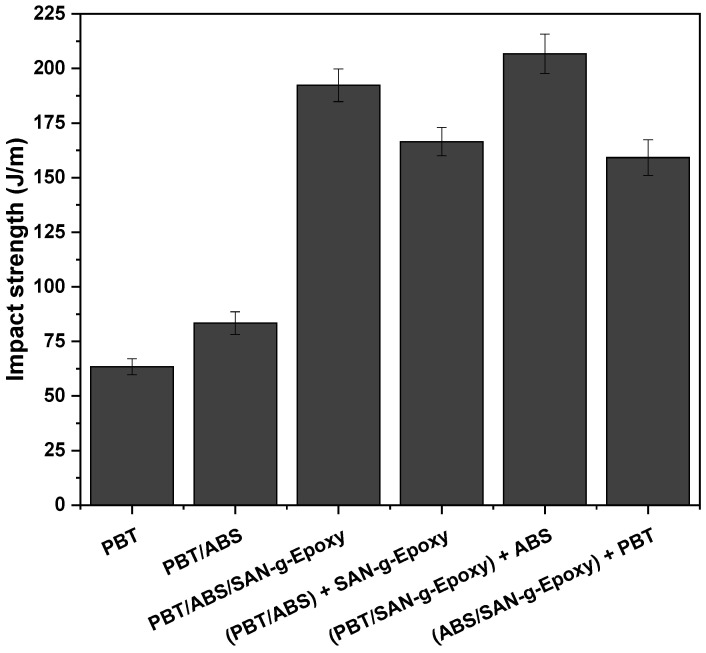
Impact strength behavior of PBT, the PBT/ABS blend, and the compatibilized systems, with the different mixing protocols.

Figure 9 shows the impact strength of neat PBT and the polymer blends, with and without the compatibilizing agent, as well as the different mixing protocols.

### 2.6. Tensile Strength

**Figure 10:**
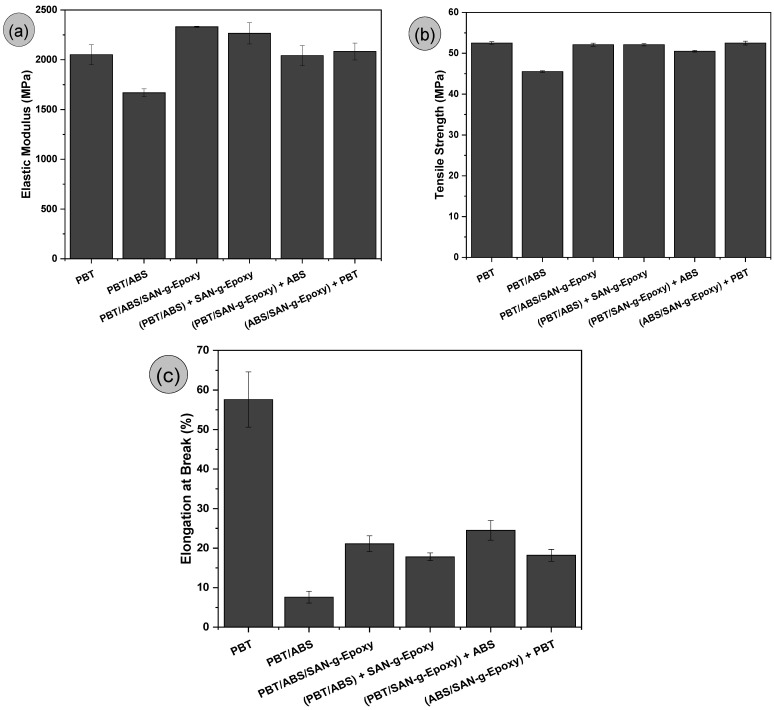
Tensile mechanical properties of pure PBT and the polymer blends, before and after compatibilization, for: (**a**) elastic modulus; (**b**) tensile strength; (**c**) elongation at break.

Figure 10a–c shows the mechanical properties under tensile loading—elastic modulus, tensile strength, and elongation at break—for pure PBT and the polymer blends.

### 2.7. Heat Deflection Temperature (HDT)

**Figure 11:**
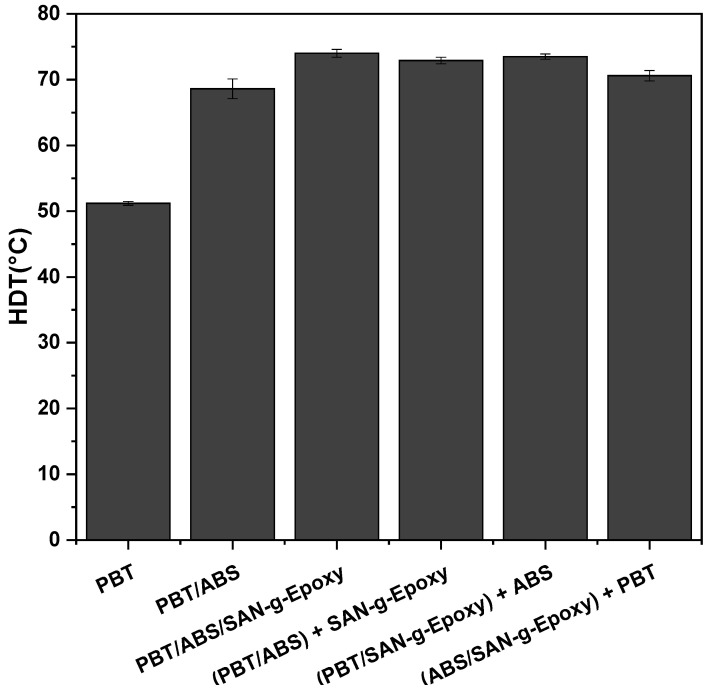
Thermomechanical stability assessed by HDT for neat PBT and PBT/ABS/SAN-g-Epoxy blends, with the different mixing sequences.

HDT is a widely used parameter to estimate the thermal resistance of polymers and polymer blends when simultaneously subjected to mechanical load and increasing temperature. This test allows for the determination of the thermal limit at which the material begins to undergo appreciable deformation under applied stress, reflecting its structural stability under service conditions [46,47]. Figure 11 presents the HDT values obtained for neat PBT and for PBT/ABS/SAN-g-Epoxy blends, considering the different mixing protocols employed.

### 2.8. Dynamic Mechanical Thermal Analysis (DMTA)

**Figure 12:**
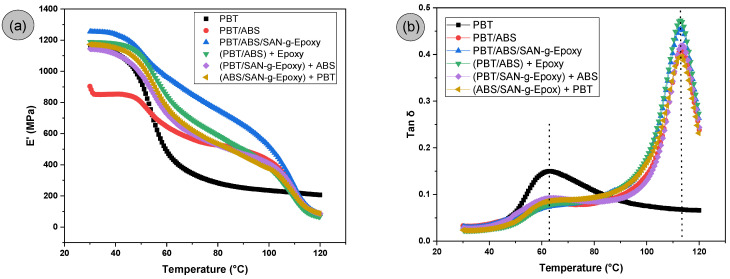
Thermomechanical behavior by DMTA for neat PBT and PBT/ABS blends, with the different mixing protocols, showing: (**a**) Storage modulus (E′); (**b**) Damping factor (tan δ).

Figure 12a,b shows the DMTA behavior of neat PBT and PBT/ABS blends, with the different mixing routes, for the storage modulus (E′) and damping factor (tan δ) as a function of temperature. The values of E’ and the glass transition temperatures corresponding to tan δ are presented in Table 1.

### 2.9. Differential Scanning Calorimetry (DSC)

**Figure 13:**
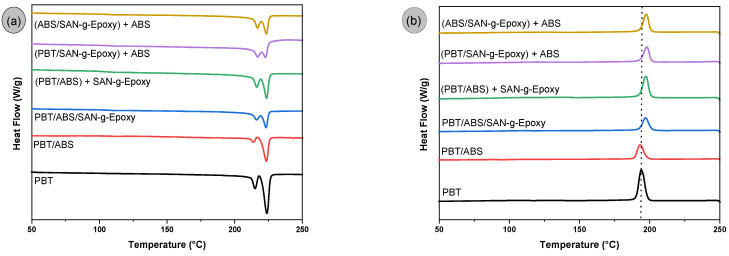
DSC curves obtained during cooling and the second heating cycle for neat PBT and polymer blends, with and without the SAN-g-Epoxy compatibilizer, for: (**a**) heating; (**b**) cooling.

Figure 13a,b shows the DSC curves obtained during cooling and the second heating cycle for neat PBT and PBT/ABS blends, with and without the SAN-g-Epoxy compatibilizer. Table 2 reports the crystallization temperatures (T_c_), melting temperatures (T_m_), and degree of crystallinity (X_c_).

## 3. Discussion

### 3.1. Torque Rheometry

Under the same processing conditions, neat PBT exhibited higher torque values than those observed for the SAN-g-Epoxy compatibilizer. This behavior can be attributed to the higher viscosity of PBT under the imposed shear conditions. On the other hand, ABS showed higher torque than PBT, i.e., 20.7 N·m compared to 16.8 N·m for the terminal torque at 10 min. Consequently, ABS presents the highest viscosity among the neat materials. When 40% ABS was added to PBT, a terminal torque of approximately 13.3 N·m was observed, which is lower than that of neat PBT. Although ABS has a higher viscosity than PBT, the torque behavior indicates low interaction between the components, resulting in a reduced viscosity response. This result demonstrates the incompatibility of the PBT/ABS system, as also reported in a previous study [48]. However, when the PBT/ABS blend was compatibilized with the reactive SAN-g-Epoxy copolymer, a significantly altered torque profile was observed.

Reactive compatibilizers are functionalized copolymers capable of promoting chemical interactions between originally immiscible phases in polymer blends. These materials contain molecular segments with affinity for one of the constituents of the blend, while reactive functional groups can chemically interact with the other component during melt processing. This mechanism contributes to stabilizing the interface between the components [49]. Canto et al. [37] investigated PBT/ABS blends compatibilized with MMA-GMA. The authors demonstrated high reactivity between the GMA functional groups and the carboxyl/hydroxyl groups of PBT, resulting in a significant increase in torque and indicating reactive compatibilization.

In the case of PBT/ABS-based systems compatibilized with SAN-g-epoxy, the compatibilization mechanism is associated with the reactivity of the epoxy groups present in the compatibilizer. During melt mixing, these groups may react with the terminal hydroxyl and/or carboxyl groups of the PBT chains, promoting interactions between the phases. Simultaneously, the SAN fraction of the compatibilizer exhibits thermodynamic affinity with the ABS phase, favoring its anchoring within these phases. The addition of 5 phr of the SAN-g-Epoxy compatibilizer to the PBT/ABS blend (60/40 wt%) promoted a significant increase in torque, resulting in the highest value among all evaluated systems. This increase indicates that, in addition to rheological changes associated with the dispersion of ABS and SAN-g-Epoxy, reactions between the functional groups likely occurred during processing, leading to an increase in torque. The temporal evolution of the torque curve provides direct evidence of this reactive compatibilization process. The PBT/ABS blend exhibited a torque of 13.3 N·m, whereas the PBT/ABS/SAN-g-Epoxy blend increased to 28.7 N·m, corresponding to a gain of 115.7%. At the same time, this value surpassed the torque of both neat PBT and ABS, indicating a more effective molecular entanglement mechanism in the PBT/ABS/SAN-g-Epoxy blend, leading to interfacial compatibilization directly reflected in the increase in torque. As shown in Figure 1, for the PBT/ABS/SAN-g-Epoxy blend, the torque begins to increase more markedly from approximately 3 min of mixing, at which point it even exceeds the value recorded for neat ABS. This behavior suggests that the initial processing time is dominated by the melting, homogenization, and plasticization of the components, whereas the subsequent increase is associated with the progression of compatibilization reactions. As the chemical interactions between the epoxy groups of SAN-g-Epoxy and the functional groups of PBT intensify, combined with the good interaction between the SAN fractions of the compatibilizer and ABS, an increase in the system viscosity occurs, resulting in higher torque performance. From a practical standpoint, the behavior of the PBT/ABS/SAN-g-Epoxy blend indicates the formation of lower interfacial tension between the components, contributing to improved adhesion between the phases and effective compatibilization. This effect is reflected in the enhanced mechanical properties discussed later, as well as in the stability of the blend morphology.

The compatibilization mechanism in the PBT/ABS blend containing SAN-g-Epoxy can be understood from the simultaneous action of thermodynamic interactions and interfacial chemical reactions that occur during melt processing (see Figure 2). The SAN segment present in SAN-g-Epoxy exhibits good affinity with the SAN phase of ABS; in other words, the similarity of part of the molecular structure favors interactions. This tendency can be interpreted based on the solubility parameters associated with the SAN constituents, approximately 31.5 (J/cm^3^)^1/2^ for acrylonitrile and 17.6 (J/cm^3^)^1/2^ for styrene [50], whose values indicate sufficient proximity to allow interaction with the corresponding segments present in ABS and SAN-g-Epoxy. In this way, the SAN portion of the compatibilizer tends to preferentially locate within the ABS phase, possibly orienting the reactive epoxy groups toward the PBT phase. Terminal hydroxyl (–OH) and carboxyl (–COOH) groups are present in PBT, which can react with the epoxy groups of SAN-g-Epoxy, forming ether groups (via hydroxyl attack) or ester groups (via carboxyl attack). As a consequence of these interactions, the PBT/ABS/SAN-g-Epoxy blend exhibits lower interfacial tension, minimizing the coalescence of dispersed phases during processing. This effect contributes to the morphological stabilization of the blend, as verified later by SEM analysis, and is positively reflected in the mechanical properties.

### 3.2. Oscillatory Rheology

Neat PBT exhibited the lowest complex viscosity values and a relatively stable profile over the analyzed frequency range, suggesting a typical Newtonian fluid behavior. This behavior indicates lower resistance to flow in the molten state, associated with the high mobility of the polymer chains under the test conditions.

The incorporation of ABS into PBT resulted in an increase in complex viscosity compared to neat PBT, indicating greater resistance to flow. This behavior differs from that observed in the torque rheometry profile, as shown in Figure 1. The apparent divergence between the results obtained from torque rheometry and complex viscosity under oscillatory conditions can be understood by considering the differences in the deformation conditions imposed on the material. In torque rheometry, the material is subjected to shear under high deformation rates during the melt mixing process. Under these conditions, the PBT/ABS blend may undergo intense deformation and elongation of the dispersed ABS particles, in addition to possible orientation of the polymer chains in the flow direction. This phenomenon tends to reduce the resistance to flow of the PBT/ABS blend, since the orientation of the phases and polymer chains facilitates molecular sliding. As a consequence, the PBT/ABS blend may exhibit lower torque than neat PBT, reflecting a lower effective viscosity under intense shear conditions typical of processing. On the other hand, in oscillatory rheology used to determine the complex viscosity (η*), the PBT/ABS blend is subjected to small deformations compared to those applied in torque rheometry. Thus, the rheological response becomes strongly influenced by the internal structure of the material, particularly by the presence of the dispersed ABS phase. The addition of ABS into PBT acts as a structural heterogeneity, increasing resistance to oscillatory deformation and resulting in complex viscosity values higher than those observed for neat PBT. Moreover, at low oscillatory frequencies, the presence of dispersed ABS domains may act as obstacles to the relaxation of PBT matrix chains, increasing the relaxation time of the system and, consequently, the measured complex viscosity.

The PBT/ABS and PBT/ABS/SAN-g-Epoxy blends exhibited curves with pseudoplastic behavior, characterized by a decrease in viscosity with increasing frequency. Nevertheless, the viscosity of the PBT/ABS blend remained lower than that observed for the compatibilized formulations, regardless of the adopted mixing protocol. This indicates that the interfacial interaction between PBT and ABS, in the absence of the compatibilizer, is relatively limited. For the PBT/ABS blends compatibilized with SAN-g-Epoxy, a more pronounced increase in complex viscosity was observed across the entire analyzed frequency range compared to both neat PBT and the uncompatibilized blend. This increase suggests the occurrence of stronger interfacial interactions between PBT and ABS in the presence of SAN-g-Epoxy, as previously discussed in the torque rheometry results. As a consequence, more stable interfacial structures are formed, which restrict molecular mobility and increase the resistance to flow of the molten system. Among the different mixing strategies, the system prepared by the simultaneous incorporation of the components (PBT/ABS/SAN-g-Epoxy) exhibited rheological behavior similar to that of the blend obtained through the strategy (PBT/ABS) + SAN-g-Epoxy, showing nearly equivalent viscosity values across the entire analyzed frequency range (0.1–600 rad/s). This result suggests that, in these two cases, the distribution of the compatibilizer at the interfaces between PBT and ABS occurs in a relatively similar manner.

On the other hand, more pronounced differences were observed at low frequencies (0.1–1 rad/s), a region particularly sensitive to the interfacial structure and morphology of the system. The blend prepared via the protocol (PBT/SAN-g-Epoxy) + ABS exhibited the highest viscosity among the compatibilized blends within this frequency range. This behavior may indicate that the pre-interaction between PBT and SAN-g-Epoxy favors the formation of a more robust interfacial region prior to the incorporation of ABS, increasing adhesion between the phases and hindering the relaxation of polymer chains at low deformation rates, resulting in a higher viscosity response. In contrast, the system prepared using the protocol (ABS/SAN-g-Epoxy) + PBT exhibited the lowest viscosity values among the compatibilized blends within the same frequency range. This behavior suggests that when the SAN-g-Epoxy compatibilizer is initially associated with the ABS phase, its action at the final interface may occur less efficiently with PBT, resulting in lower restriction of chain mobility and, consequently, lower resistance to flow at low deformation rates.

The increase in complex viscosity (η*) in compatibilized polymer blends is strongly related to structural changes in the morphology of the dispersed phase and at the interface between the polymers [51,52]. In uncompatibilized PBT/ABS blends, the morphology tends to present relatively large ABS phase domains and a weakly adhered interface, as verified later by SEM analysis. Under these conditions, during oscillatory deformation, the PBT and ABS phases can slide more easily over one another, allowing greater chain mobility and resulting in lower complex viscosity values compared with compatibilized systems, especially in the low-frequency region. With the incorporation of SAN-g-Epoxy into PBT/ABS, a more efficient reactive compatibilization process occurs. As previously discussed in the torque rheometry results, the epoxy groups present in the compatibilizer can react with terminal groups of PBT, while the SAN segments exhibit chemical affinity with the ABS phase. This mechanism favors the formation of a more stable morphology between the phases. As a consequence, interfacial tension is reduced, promoting morphological refinement of the ABS phase. This refinement results in smaller ABS domains that are more uniformly distributed within the PBT matrix, significantly increasing the interfacial area between the phases. The greater contact area and stronger interfacial interactions restrict the relative motion between the phases during rheological deformation. As a result, energy dissipation becomes more pronounced, leading to an increase in complex viscosity.

Therefore, in the PBT/ABS/SAN-g-Epoxy system and the different mixing protocols, the increase in complex viscosity can be interpreted as an indicator of improved morphology in the compatibilized blends, which was further corroborated by SEM analysis. This reflects the formation of a more stable interface, greater adhesion between the phases, and a reduction in the size of the dispersed phase, factors that contribute to the formation of polymer blends with greater resistance to flow.

As shown in Figure 4a,b, in general, PBT and the polymer blends exhibited a progressive increase in G′ and G″ with increasing frequency, during which both the elastic and viscous responses became more pronounced. For neat PBT, the G′ and G″ values were lower than those observed for the polymer blends, particularly compared to the SAN-g-Epoxy compatibilized systems. In addition, PBT displayed a predominance of the loss modulus over the storage modulus across nearly the entire frequency range, indicating dominant viscous behavior. With the incorporation of ABS, an increase in both G′ and G″ was observed in the PBT/ABS blend compared to neat PBT. This increase can be attributed to the addition of ABS, which alters the relaxation dynamics of PBT. The presence of ABS as a dispersed phase contributed to forming additional restrictions to the movement of PBT chains, requiring more energy to accommodate the deformation imposed during the oscillatory test. As a result, the PBT/ABS blend exhibits a greater elastic and viscous contribution compared to neat PBT.

In the SAN-g-Epoxy compatibilized blends and across the different mixing protocols, the G′ and G″ values were even higher compared to the base PBT/ABS system, following the same trend observed for complex viscosity. This finding suggests that the presence of SAN-g-Epoxy promotes stronger interfacial interactions between PBT and ABS, resulting in greater molecular entanglement between the phases and reinforcing the interface. The formation of an interface with higher adhesion between PBT and ABS tends to limit the independent relaxation of chains in each phase, increasing the system’s ability to store and dissipate mechanical energy during oscillatory deformation. A particularly relevant aspect was observed in the low-frequency region, approximately between 0.1 and 1 rad/s, where a more pronounced plateau was detected for the compatibilized blends, especially in the storage modulus (G′). This behavior indicates that, in this frequency range, the system exhibits slower molecular relaxation, suggesting the presence of more strongly adhered interfacial structures and reinforcing the effectiveness of SAN-g-Epoxy in compatibilizing PBT/ABS. Consequently, the PBT/ABS/SAN-g-Epoxy blends and the different mixing protocols suggest that the phases do not respond completely independently to deformation. In other words, the response arises from the adhered ensemble of the phases, where the interface plays an important role in stress transmission.

The effect of the mixing protocol used had a subtle impact on the G′ and G″ values, specifically in the range of 0.1 to 1 rad/s. The (PBT/SAN-g-Epoxy) + ABS route tended to exhibit higher G′ values, suggesting the formation of an interface with a greater degree of entanglement, which may have contributed to the superior impact performance discussed later. The PBT/ABS/SAN-g-Epoxy and (PBT/ABS) + SAN-g-Epoxy protocols showed similar trends, with overlapping curves. In contrast, when using the (ABS/SAN-g-Epoxy) + PBT protocol, the effect on viscoelastic stiffness at low frequencies tended to be less pronounced compared to the other compatibilized blends.

### 3.3. Fourier Transform Infrared Spectroscopy (FTIR)

In Figure 5a, neat PBT exhibited a strong band at 1706 cm^−1^, attributed to the stretching of the carbonyl group (C=O) of the ester. In the region between 1500 and 1600 cm^−1^, small bands are observed, associated with the stretching vibrations of the aromatic ring present in the terephthalic unit. Additional bands appear between 1000 and 1260 cm^−1^, related to C–O–C stretching of the ester group. In the 2850–2960 cm^−1^ region, bands assigned to the C–H stretching of methylene groups (–CH_2_–) from the aliphatic butylene segment are identified. Moreover, absorptions in the 725–870 cm^−1^ range are associated with out-of-plane deformations of the aromatic ring. These bands are consistent with the literature [53,54]. For ABS, bands are observed between 2920 and 2850 cm^−1^ (aliphatic C–H stretching), 2235–2240 cm^−1^ (C≡N stretching of acrylonitrile, see Figure 5b), 1638 cm^−1^ (C=C stretching of butadiene units), 1600–1490 cm^−1^ (C=C vibrations of the styrene aromatic ring), 966 cm^−1^ (out-of-plane deformation of the C=C of butadiene (trans-1,4)), and 910 cm^−1^ (vibration associated with 1,2-butadiene) [55]. The FTIR spectra of the PBT/ABS and PBT/ABS/SAN-g-Epoxy blends, as well as the different mixing protocols, tended to show a superposition of the PBT and ABS bands, with emphasis on the appearance of the acrylonitrile C≡N stretching band at 2236 cm^−1^, as shown in Figure 5b. This indicates that ABS is accommodated between the PBT chains, which is essential for the toughening process, as further evidenced in the impact strength results.

The main modification in the spectra is associated with the band at 1706 cm^−1^ of the carbonyl group (C=O), as shown in Figure 5c. The PBT/ABS blend exhibited a slight shift of the carbonyl band relative to neat PBT. However, a significant reduction in the absorption intensity was observed, indicating limited physical interaction between PBT and ABS, which is consistent with the torque rheometry results and the mechanical properties discussed later. In this case, only the additive effect of each component prevailed, i.e., 70% PBT with 30% ABS, resulting in a lower amount of carbonyl groups in the formulation due to the reduced PBT content. When the PBT/ABS blend was compatibilized with SAN-g-Epoxy, a slight shift of the carbonyl band was also observed. However, an increase in the absorption intensity compared to neat PBT was detected, reinforcing the suggestion of reactive compatibilization, as previously indicated by torque rheometry and discussed in the mechanism illustrated in Figure 2. The significant increase in the carbonyl band intensity in the PBT/ABS/SAN-g-Epoxy blend, particularly surpassing that of neat PBT, is a strong indication of chemical reactions involving the opening of the epoxy ring in the compatibilizer with the terminal groups of PBT, especially –COOH and –OH. In systems containing epoxy groups, the most probable reaction occurs with the terminal –COOH and –OH groups of PBT, as also reported in similar work with PET [56]. Apparently, as the carbonyl band intensity increased, the formation of ester groups predominated. In other words, when the epoxy groups of SAN-g-Epoxy react with carboxyl (–COOH) groups of PBT, ring opening occurs, followed by the formation of new ester bonds, potentially generating PBT-g-SAN structures. This process increases the effective number of C=O groups, which is reflected in the enhanced intensity of the carbonyl band in the FTIR spectra. Regarding the different mixing protocols, the (PBT/SAN-g-Epoxy) + ABS route exhibited the highest carbonyl band intensity, suggesting the highest level of reactivity. The pre-mixing of PBT with SAN-g-Epoxy favors reactive compatibilization between the components, generating a greater number of carbonyl groups. In contrast, pre-mixing ABS with SAN-g-Epoxy caused a certain level of inhibition of subsequent reactivity with PBT, resulting in a lower carbonyl band intensity. The (PBT/ABS) + SAN-g-Epoxy protocol maintained an intensity similar to that of neat PBT, indicating that pre-mixing PBT with ABS slightly hindered the reactivity upon subsequent addition of the compatibilizer.

### 3.4. Scanning Electron Microscopy (SEM)

For pure PBT, as shown in Figure 6a, the fracture surface exhibited a relatively homogeneous appearance with moderate signs of plastic deformation, suggesting some degree of deformation prior to failure. Slightly deformed regions and some smooth surfaces were observed, characterizing intermediate behavior between ductile and brittle fracture. However, the absence of fibrillated regions indicates that deformation was limited, corroborating the impact strength results discussed later. For the PBT/ABS blend, shown in Figure 6b, the fracture surface displayed features typical of multiphase systems, with an irregular fracture pattern and coarse morphology. Dispersed ABS domains within the PBT matrix were observed, along with voids on the fracture surface. This morphology suggests that during fracture, some of the dispersed ABS particles were pulled out from the PBT matrix, forming voids. This phenomenon indicates limited interfacial adhesion between PBT and ABS, allowing cracks to propagate preferentially along the interfacial regions. Consequently, energy dissipation during fracture is low, preventing extensive plastic deformation of the PBT and confirming the low impact performance reported later.

The PBT/ABS/SAN-g-Epoxy blend and the different mixing protocols exhibited a fracture morphology with significant changes compared to the uncompatibilized blend. The plastic deformation mechanism was more pronounced, with a more regular morphology and no interfacial delamination. This suggests that during the impact test, crack propagation occurred more slowly. Additionally, a greater number of ABS particles were observed, well-dispersed and strongly adhered within the PBT matrix, indicating improved interfacial strength. This behavior can be attributed to the action of the SAN-g-Epoxy compatibilizer in the PBT/ABS system, which tends to reduce interfacial tension between the phases and enhance adhesion at the interface, facilitating better stress distribution and dissipation. Clearly, there was significant refinement of the ABS particles within the PBT, demonstrating that SAN-g-Epoxy effectively reduced interfacial tension and prevented coalescence, resulting in a more stable and refined morphology. This observation aligns with the discussion presented regarding complex viscosity.

In Figure 7a, the PBT/ABS blend exhibited a dispersed ABS phase with relatively large and heterogeneous particles. The large size of these voids indicates that significant coalescence of ABS droplets occurred during processing. This phenomenon is associated with the limited interfacial affinity between PBT and ABS, reducing the stability of ABS particles under shear conditions during processing. Consequently, small ABS droplets tend to merge, forming larger domains throughout the blend. The presence of large ABS particles suggests that interfacial tension forces predominated over shear-induced breakup during processing, favoring coalescence rather than fine dispersion. Furthermore, the distribution of ABS particles within the PBT matrix was irregular, with some regions densely populated by ABS and others almost devoid. This heterogeneous morphology, characterized by larger ABS domains, directly impacted the mechanical properties of the PBT/ABS blend, particularly leading to low impact strength.

The incorporation of SAN-g-Epoxy into PBT/ABS significantly affected the morphology, as shown in Figure 7b for the simultaneous mixing approach. A clear reduction in the size of ABS particles within the PBT matrix was observed, suggesting that the compatibilizer effectively reduced the interfacial tension between the phases. As previously discussed, the epoxide groups in SAN-g-Epoxy can interact with the terminal groups of PBT, while the SAN fraction exhibits thermodynamic affinity for the SAN phase of ABS. This effect helps lower the interfacial energy and stabilize the ABS particles, hindering coalescence during shear in the molten blend and promoting the formation of smaller, well-dispersed particles [57,58]. Regarding the different mixing sequences of SAN-g-Epoxy in PBT/ABS, the morphologies were generally similar, showing only minor variations. The PBT/ABS/SAN-g-Epoxy, (PBT/ABS) + SAN-g-Epoxy, and (ABS/SAN-g-Epoxy) + PBT protocols displayed ABS particles with a more rounded shape, good distribution, and close interparticle spacing. Notably, the most stable morphology was observed for the (PBT/SAN-g-Epoxy) + ABS sequence, where particles were well-dispersed and interconnected, as shown in Figure 7d. Performing the pre-mixing of PBT with SAN-g-Epoxy appears to favor its migration to the interface upon subsequent addition of ABS, enhancing interfacial compatibilization.

As shown in Figure 8, the PBT/ABS blend exhibited an average ABS particle size of 4.34 ± 1.21 µm, indicating a heterogeneous distribution with large particles. Compatibilized blends showed a pronounced refinement of the ABS phase, demonstrating the effectiveness of SAN-g-Epoxy in interacting with the phases and preventing coalescence. The average particle sizes were similar: PBT/ABS/SAN-g-Epoxy (0.48 ± 0.1 µm), (PBT/ABS) + SAN-g-Epoxy (0.54 ± 0.1 µm), (PBT/SAN-g-Epoxy) + ABS (0.47 ± 0.07 µm), and (ABS/SAN-g-Epoxy) + PBT (0.52 ± 0.08 µm). In PBT toughening processes, it has been shown that maximum toughness occurs in the ranges of 0.430–0.506 µm [20] and 0.240–0.485 µm [59]. The results for the PBT/ABS/SAN-g-Epoxy blends and their mixing sequences are consistent with literature reports, which likely contributed to the excellent mechanical performance discussed later.

### 3.5. Impact Strength

Pure PBT exhibited an impact strength of 63.4 J/m, while the incorporation of ABS increased this value to 83.4 J/m, corresponding to a gain of 31.5%. Although this improvement is moderate considering the use of 40% ABS in the formulation, some toughening effect was achieved. The addition of the SAN-g-Epoxy compatibilizer to PBT/ABS led to more significant increases in impact strength, depending on the mixing protocol employed. The simultaneous PBT/ABS/SAN-g-Epoxy sequence showed an impact strength of 192.3 J/m, approximately three times higher than that of pure PBT. This performance indicates that the addition of SAN-g-Epoxy enhanced the interaction between PBT and ABS, reinforcing the interface of the polymer blend. This reinforcement was crucial for improving stress transfer between the phases and achieving higher energy dissipation in the matrix. As observed in SEM, the morphological changes in PBT/ABS/SAN-g-Epoxy evidenced interfacial adhesion and good distribution of the ABS phase, favoring an effective toughening mechanism.

The analysis of the different mixing sequences reveals that the processing protocol influences the impact strength, suggesting it affects the effectiveness of reactive compatibilization. Previous studies [60,61] indicate that the sequence of compatibilizer incorporation during the processing of polymer blends can affect the material’s impact strength. On the other hand, this factor generally does not cause significant changes in the mechanical properties evaluated under tensile loading, which typically remain similar or close regardless of the mixing order employed. In Figure 9, when the compatibilizer SAN-g-Epoxy was initially blended with PBT, forming the composition (PBT/SAN-g-Epoxy) + ABS, the highest impact strength was observed (206.7 J/m). This result indicates that the pre-interaction between SAN-g-Epoxy and PBT likely promoted reactive compatibilization, forming PBT-g-SAN. Subsequently, during the addition of ABS, the PBT-g-SAN system favored greater interfacial entanglement with ABS, promoting a more stable morphology and improved interfacial adhesion, which likely contributed to higher energy dissipation. This behavior aligns with the findings from complex viscosity and FTIR analyses. On the other hand, when SAN-g-Epoxy was added after the pre-mixing of PBT/ABS, the impact strength decreased to 166.5 J/m, a value lower than those obtained for PBT/ABS/SAN-g-Epoxy and (PBT/SAN-g-Epoxy) + ABS. In this case, the interaction of the compatibilizer may have been limited due to reduced accessibility to the already formed interfaces between PBT and ABS. Consequently, interfacial interaction was less efficient, resulting in lower energy dissipation during fracture. A similar situation was observed for the composition (ABS/SAN-g-Epoxy) + PBT, which exhibited an impact strength of 159.2 J/m. Here, by pre-mixing SAN-g-Epoxy with ABS, the chemical affinity between the SAN fractions of both components likely led to good adhesion, limiting the availability of epoxide groups to subsequently react with PBT. As a result, the efficiency of compatibilization at the PBT/ABS interface was reduced, leading to a smaller improvement in the toughness of this blend.

In terms of impact strength, the values obtained between 159.2–207 J/m for the PBT/ABS/SAN-g-epoxy blends exceeded those described by Liu et al. [62], as well as showing values similar to those reported by Sun et al. [63]. Furthermore, the results are important from a technological standpoint, as they are higher than those of engineering blends such as PA6/ABS/SAM [44] and PA6/ABS/MM-MA [64], and close to the values reported for PA6/AES/MMA [65]. However, the results of the PBT/ABS/SAN-g-epoxy blends were lower than those reported in [66] for PBT/ABS-g-GMA blends. This can possibly be explained by the high degree of reactivity of ABS-g-GMA, considering that it contained 8% grafted GMA, which enhanced the interaction between PBT and ABS.

### 3.6. Tensile Strength

Pure PBT exhibited an elastic modulus of approximately 2050 MPa, a value similar to that reported in [31]. The PBT/ABS blend showed a reduction in elastic modulus of 18.6% compared to pure PBT. According to the ABS manufacturer, the tensile elastic modulus is 1900 MPa. Since the PBT/ABS blend performed worse than the individual polymers, this confirms the low compatibility between the components. This indicates that during the test, premature fracture occurred at the PBT/ABS interfacial region, leading to the lower elastic modulus. The incorporation of SAN-g-Epoxy into PBT/ABS improved the elastic modulus relative to the uncompatibilized blend. In the formulation prepared by simultaneous mixing (PBT/ABS/SAN-g-Epoxy), the highest modulus value was observed (2230.9 MPa), even surpassing pure PBT by 8.8%. This result supports the hypothesis that SAN-g-Epoxy promoted compatibilization, as previously evidenced in complex viscosity and FTIR analyses, enhancing the efficiency of mechanical load transfer and resulting in greater system stiffness.

The variations observed in the elastic modulus of PBT/ABS/SAN-g-Epoxy blends prepared via different mixing sequences can be mainly attributed to subtle changes in morphology and the extent of interfacial interactions developed during processing. Although all formulations have the same overall composition, the order of SAN-g-Epoxy incorporation can influence how the compatibilizer is distributed within the PBT/ABS system, and consequently, how stress is transferred through the material. The sequences PBT/ABS/SAN-g-Epoxy and (PBT/ABS) + SAN-g-Epoxy were the most effective in optimizing elastic modulus results, both falling within the experimental error margin. Since these values exceeded that of pure PBT, it is reasonable to suggest that SAN-g-Epoxy also contributed to increased stiffness. In this case, part of the SAN-g-Epoxy likely migrated to the PBT/ABS interface, while another fraction remained dispersed in the PBT matrix, producing a favorable response in the elastic modulus. The protocols (PBT/SAN-g-Epoxy) + ABS and (ABS/SAN-g-Epoxy) + PBT showed elastic modulus values comparable to pure PBT, which is technologically significant. That is, these formulations maintain stiffness while enhancing impact performance, resulting in materials with a balanced combination of properties. Apparently, when SAN-g-Epoxy is pre-mixed with either PBT or ABS, forming (PBT/SAN-g-Epoxy) + ABS and (ABS/SAN-g-Epoxy) + PBT, the elastic modulus tends to slightly decrease compared to the other compatibilized blends. This suggests increased molecular entanglement during mixing, which may have hindered the migration of part of the SAN-g-Epoxy to form a third dispersed phase in the PBT matrix, with predominance at the interfacial region.

In Figure 10b, the effect of compatibility in the PBT/ABS/SAN-g-Epoxy blends and the different mixing sequences is clearly evident, with recovery of tensile strength. Pure PBT exhibited a tensile strength of 52.5 MPa, while the PBT/ABS blend declined to 45.5 MPa. Since tensile strength is measured in the plastic deformation regime, interfacial adhesion has a strong influence on this property. The lower performance of PBT/ABS can be attributed to limited interfacial interaction between the components, leading to the formation of microvoids at the interface. Consequently, these act as stress concentrators, resulting in premature fracture and reduced tensile strength. The PBT/ABS/SAN-g-Epoxy blend and the different mixing sequences maintained tensile strength values similar to those of pure PBT, within the experimental error margin. The improvement in tensile strength for the compatibilized blends is mainly associated with the reactive compatibilization effect promoted by SAN-g-Epoxy. The epoxy groups in SAN-g-Epoxy can interact with the terminal hydroxyl or carboxyl groups of PBT, forming bonds at the interface between the phases. At the same time, the SAN chains exhibit affinity with the SAN-rich phase present in ABS. This dual affinity promotes the formation of more stable interfacial adhesion, enhancing stress transfer and increasing tensile strength. This behavior is supported by the increased viscosity observed in rheological tests and the higher impact strength, indicating more efficient interfacial interaction. Regarding the different mixing protocols, no significant changes were observed for this property with selective distribution of SAN-g-Epoxy, with only maintenance of the results. The tensile strength results observed are comparable to those reported in [67] for PBT/ABS blends compatibilized with methyl methacrylate–glycidyl methacrylate–ethyl acrylate (MGE). However, the tensile strength of the PBT/ABS/SAN-g-epoxy blends is higher than that reported by Lu and Qu [68] for PBT/SEBS-g-MA blends and by Yang et al. [27] for PBT/HIPS-g-GMA blends. This indicates that, when an effective compatibilizer is used in PBT/ABS blends, such as SAN-g-epoxy, the tensile mechanical performance is superior compared to conventional impact modifiers.

Pure PBT exhibited an elongation at break of approximately 57.6%, whereas the PBT/ABS blend dropped sharply to 7.6%. Being a homogeneous material, PBT allows its chains to align in the direction of the applied load, resulting in a high elongation response. In contrast, the PBT/ABS blend is a heterogeneous system whose behavior strongly depends on interfacial adhesion, which, as previously shown, is low between the phases, leading to poor elongation at break. The compatibilized blends exhibited increased ductility under tensile loading compared to the uncompatibilized PBT/ABS system, indicating improved compatibility. The (PBT/ABS) + SAN-g-Epoxy and (ABS/SAN-g-Epoxy) + PBT blends showed similar elongation results. Greater ductility was observed for the PBT/ABS/SAN-g-Epoxy and (PBT/SAN-g-Epoxy) + ABS protocols, which also corresponded to the highest impact strength values. These compositions demonstrated superior performance relative to the others, achieving a better balance of mechanical properties.

### 3.7. Heat Deflection Temperature (HDT)

Neat PBT exhibited a relatively low HDT value of around 52.8 °C, a result similar to that reported by Colombo et al. [69]. The incorporation of ABS led to a significant increase in this parameter, regardless of the presence of a compatibilizer in the formulation. Specifically, the addition of 40 wt% ABS to PBT raised the HDT to 68.6 °C, corresponding to an approximate 30% increase compared to neat PBT. This result indicates that ABS effectively contributes to improving the thermomechanical performance of PBT, a relevant aspect considering that low HDT is one of the main limitations of PBT. Similar findings were observed by Ferreira et al. [31], who evaluated the effect of different types of ABS on the mechanical and thermomechanical properties of PBT. According to the authors, the PBT/ABS (60/40 wt%) blend maintained impact strength while showing a reduction in elastic modulus and tensile strength. Conversely, a significant increase in HDT was observed for this composition, attributed to the presence of acrylonitrile in the SAN phase of ABS, which tends to enhance the thermomechanical stability of the material.

The incorporation of 5 phr of the reactive compatibilizer SAN-g-Epoxy resulted in an additional increase in HDT, reaching 74 °C for the blend prepared via simultaneous mixing of PBT/ABS/SAN-g-Epoxy, corresponding to an increase of approximately 5.4 °C compared to the PBT/ABS blend. This behavior may be associated with the increased total SAN content in the formulation, due to the combined contribution of the SAN present in ABS and that from the compatibilizer, which tends to favor the enhancement of the thermomechanical response evaluated by HDT. The compositions obtained through different mixing sequences, (PBT/ABS) + SAN-g-Epoxy (72.9 °C), (PBT/SAN-g-Epoxy) + ABS (73.5 °C), and (ABS/SAN-g-Epoxy) + PBT (70.6 °C), also exhibited HDT values higher than those observed for the base PBT/ABS blend. Compared to neat PBT, the increases in thermomechanical stability of the compatibilized blends were even more pronounced, exceeding 17 °C, regardless of the processing route adopted. It is observed that the (PBT/ABS) + SAN-g-Epoxy and (PBT/SAN-g-Epoxy) + ABS routes produced very similar results, with no significant variations. On the other hand, the (ABS/SAN-g-Epoxy) + PBT route showed a slightly lower HDT compared to the other compatibilized formulations. Considering that SAN-g-Epoxy contains a SAN fraction with high chemical affinity for the SAN present in ABS, it is possible that the pre-mixing of these components favored preferential interactions between them, reducing the extent of reactive interactions with PBT. Consequently, a lower degree of interfacial anchoring may have occurred, resulting in a slight reduction in HDT.

From a technological perspective, the results obtained for the PBT/ABS/SAN-g-Epoxy blends, as well as for the different mixing routes employed, demonstrate a significant increase in thermomechanical stability as assessed by HDT. This enhancement expands the potential applications of PBT in scenarios where the material is exposed to elevated temperatures during use, including automotive components, electrical equipment housings, electronic devices, and various structural elements used in engineering applications.

### 3.8. Dynamic Mechanical Thermal Analysis (DMTA)

The storage modulus (E′) represents the material’s ability to store elastic energy when subjected to oscillatory deformation and is, therefore, an indicator of the system’s stiffness over the temperature range [70,71]. In Figure 12a, within the range of 30 to 50 °C, a plateau region was observed for both neat PBT and the polymer blends, regardless of compatibilization, indicating the glassy region and characterizing rigid behavior. Fu et al. [72] also observed this event for PBT in the DMTA. Neat PBT exhibited a storage modulus of approximately 1156.7 MPa, a value higher than that measured for the PBT/ABS blend, which was 883.5 MPa. In the 30 to 50 °C range, the simultaneous PBT/ABS/SAN-g-Epoxy blend showed the highest E′ performance, with increases of 8.4% and 42% compared to neat PBT and PBT/ABS, respectively. This finding supports the hypothesis that SAN-g-Epoxy acted as a compatibilizing agent for PBT/ABS, leading to improved stress transfer and distribution between phases, which undoubtedly enhanced E′. The (PBT/ABS) + SAN-g-Epoxy, (PBT/SAN-g-Epoxy) + ABS, and (ABS/SAN-g-Epoxy) + PBT protocols maintained values similar to neat PBT, demonstrating the preservation of matrix stiffness, while exceeding the PBT/ABS blend by more than 30% in E′. These results are consistent with the tensile elastic modulus data, which indicated that SAN-g-Epoxy improved the stiffness of the PBT/ABS blend for all compositions.

For neat PBT, it was observed that approaching 60 °C (Figure 12a) there is a pronounced decrease in the storage modulus, which is attributed to the α-relaxation and associated with the glass transition temperature (T_g_) [73]. This behavior results from the increased segmental mobility in the amorphous phase, leading to a gradual transition from rigid behavior to a more flexible viscoelastic response. To better visualize the glass transition temperatures of the PBT/ABS and PBT/ABS/SAN-g-Epoxy blends and the mixing protocols, the maximum peak is shown in Figure 12b. Due to the difference between the glass transition temperatures of PBT and ABS, the thermomechanical behavior of the polymer blends tends to exhibit two distinct relaxation regions. The first is associated with the T_g_ of PBT, while the second is related to the T_g_ of ABS, around 113 °C. The DMTA results obtained in this study are similar to those reported by Liu et al. [74] for PBT/ASA systems compatibilized with epoxy resin.

In the PBT/ABS blend, the storage modulus profile is influenced by the presence of the ABS phase (Figure 12a), whose glass transition occurs at a significantly higher temperature. Thus, after the glass transition of PBT (63.2 °C), part of the system stiffness is still maintained due to the contribution of the SAN-rich phase of ABS, which remains in the glassy state within this temperature range. This effect results in a less abrupt reduction of E′ for the polymer blends compared to neat PBT, indicating that the presence of ABS acts as a structural component that partially limits PBT chain mobility. The PBT/ABS/SAN-g-Epoxy blend exhibited the highest E′ stability until approaching the glass transition, suggesting better preservation of rigidity upon heating. The mixing routes (PBT/ABS) + SAN-g-Epoxy, (PBT/SAN-g-Epoxy) + ABS, and (ABS/SAN-g-Epoxy) + PBT tend to show higher values or a slower decline with increasing temperature, particularly in the 60–80 °C range. This effect may be associated with improved interfacial interactions promoted by the reactive compatibilizer SAN-g-Epoxy in the PBT/ABS blend, as observed in SEM. The epoxy groups can react or interact with PBT terminal groups, while the SAN fraction has affinity for the ABS phase, favoring a more stable interface between the phases. Consequently, more efficient stress transfer occurs between the PBT and ABS domains, generating additional restriction to molecular mobility, which helps preserve the material’s rigidity at intermediate temperatures.

### 3.9. Differential Scanning Calorimetry (DSC)

The DSC analysis allowed for evaluating how the incorporation of ABS and the SAN-g-Epoxy compatibilizer influenced the crystallization and melting behavior of PBT. Neat PBT exhibited two crystalline melting peaks (T_m_^1^ and T_m_^2^), located approximately at 215 °C and 223.8 °C, as also reported in the literature [28]. The presence of two endothermic events is often associated with populations of crystals with different degrees of perfection. Less organized crystals corresponding to T_m_^1^ melt at lower temperatures, while thicker, more perfect crystals require higher thermal energy to melt, as observed for T_m_^2^. In the PBT/ABS and PBT/ABS/SAN-g-Epoxy blends, the crystalline melting temperatures remained virtually unchanged compared to neat PBT, maintaining both peaks within the same temperature range. Minor variations in T_m_^1^ and T_m_^2^ were not significant. The same trend was observed for the different mixing routes regarding the crystalline melting temperature, showing only slight variations. These results related to T_m_^1^ and T_m_^2^ suggest that ABS and SAN-g-Epoxy did not significantly alter the thermal stability of PBT crystals.

Regarding the crystallization temperature (T_c_), it was observed that the PBT/ABS blend exhibited a value similar to that of neat PBT (194 °C), indicating that the presence of ABS alone does not have a significant effect on the crystallization rate. However, when the PBT/ABS blend was compatibilized with SAN-g-Epoxy, a slight increase in Tc of approximately 3–4 °C was observed. Although the increase in T_c_ is modest, a slight acceleration of the crystallization process was noted. This behavior may indicate that the presence of the compatibilizer promotes interfacial modifications that favor the onset of crystallization at slightly higher temperatures. Altering the selective distribution routes of SAN-g-Epoxy in PBT/ABS did not result in significant changes compared to the simultaneous PBT/ABS/SAN-g-Epoxy blend. Similar results for the crystallization temperature were also reported in the literature [75], in PBT/AES blends compatibilized with methyl methacrylate (MMA)/glycidyl methacrylate (GMA)/ethyl acrylate (EA) terpolymers (MGEs).

The degree of crystallinity of neat PBT was around 22.7%, a value close to that reported by Ferreira et al. [76]. Regarding crystallinity, the PBT/ABS blend showed an increase compared to neat PBT, suggesting a nucleating effect. As ABS represents a structural heterogeneity dispersed within PBT, it possibly acted as energetically favorable regions for the onset of PBT crystal nucleation, thereby increasing the crystalline fraction. On the other hand, when the simultaneous PBT/ABS/SAN-g-Epoxy blend was prepared, the degree of crystallinity remained close to that of neat PBT, at 22.1%. The different mixing protocols maintained the degree of crystallinity in the range of 22–27.6%, values similar to or slightly higher than neat PBT, which likely contributed to preserving a high level of elastic modulus, as observed in tensile testing and DMTA. However, the reduction in X_c_ for the compatibilized blends compared to PBT/ABS, regardless of the SAN-g-Epoxy feeding route, was possibly due to the higher viscosity, as observed in oscillatory rheology. According to observations by Hage et al. [77] for PBT/ABS blends, increased viscosity reduces molecular mobility during crystallization, decreasing the likelihood of forming stable crystals.

The DSC results for the PBT/ABS/SAN-g-Epoxy blends and the different mixing sequences are noteworthy, as they did not significantly alter the PBT melting pattern but caused slight modifications in the crystallization kinetics and the crystalline fraction formed. This is important because it preserves the thermal parameters while providing improvements in mechanical performance, suggesting the potential of PBT for more demanding applications.

## 4. Materials and Methods

### 4.1. Materials

The polymer matrix used in this study was poly(butylene terephthalate) (PBT), commercial grade Crastin S600F10 NC010, supplied by DuPont (Wilmington, DE, USA), with a melt flow index of 11 g/10 min, determined according to ISO 1133 [78]. As the dispersed phase for impact modification, poly(acrylonitrile–butadiene–styrene) (ABS), grade Terluran HI-10, supplied by BASF (Ludwigshafen, Germany), was used. This material has a melt flow index of 5.5 cm^3^/10 min (220 °C/10 kg, ISO 1133), an Izod impact strength of 410 J/m (ASTM D256 [79]), an elastic modulus of 1900 MPa (ISO 527-1/-2 [80,81]), and a heat deflection temperature (HDT) of 96 °C under a load of 1.82 MPa (ISO 75-1/-2 [82,83]). As a compatibilizing agent, a styrene–acrylonitrile copolymer functionalized with epoxy groups (SAN-g-Epoxy) was used, identified by the code KS-05 and supplied by Coace Chemical Company Limited (Xiamen, China). The material presents an epoxy group content grafted onto the polymer chain between 2 and 4 wt%. In addition, the compatibilizer has a melt flow index in the range of 22 to 28 g/10 min, determined at 200 °C under a load of 5 kg.

### 4.2. Extrusion Processing

Prior to extrusion processing, the materials were subjected to a controlled drying procedure to remove residual moisture. PBT was dried at 80 °C, while ABS and the SAN-g-epoxy compatibilizer were conditioned at 60 °C, all in a vacuum oven (400–500 mmHg) for a period of 24 h. This procedure is particularly important to prevent hydrolytic degradation during processing, especially in the case of PBT, which is more sensitive to the presence of moisture at elevated temperatures [84]. The PBT/ABS blend was prepared using a composition of 60 wt% PBT and 40 wt% ABS. The selection of the PBT/ABS (60/40 wt%) ratio was based on specific literature [31], which demonstrated that this composition represents an optimized condition for increasing the heat deflection temperature (HDT) without causing significant detriment to the mechanical properties. To investigate the influence of the compatibilizer, the same PBT/ABS ratio was maintained, with the SAN-g-Epoxy copolymer incorporated at a proportion of 5 parts per hundred resin (phr). The adopted compatibilizer content falls within the range typically reported in studies focused on the compatibilization of polymer blend systems [33,85]. In order to investigate the influence of SAN-g-Epoxy on the compatibilization process of the PBT/ABS blend, different compatibilizer incorporation strategies were adopted. Thus, distinct mixing sequences were evaluated, as described below:

➢ PBT/ABS/SAN-g-Epoxy: Indicates that all components of the formulation were simultaneously added to the extruder during processing;
➢ (PBT/ABS) + SAN-g-Epoxy: Indicates that, in an initial step, PBT was previously processed with ABS by extrusion, forming a pre-blend. Subsequently, this material was subjected to a second processing step, in which SAN-g-Epoxy was added to the system;
➢ (PBT/SAN-g-Epoxy) + ABS: Indicates that, in an initial step, PBT was previously processed with the SAN-g-Epoxy compatibilizer by extrusion, forming a pre-blend. Subsequently, this material was subjected to a second processing step, in which ABS was incorporated into the system;
➢ (ABS/SAN-g-Epoxy) + PBT: Indicates that, initially, ABS was processed together with the SAN-g-epoxy compatibilizer in a first extrusion step, forming a pre-blend. Subsequently, this material was reprocessed in a second step, in which PBT was incorporated into the mixture.

Neat PBT, the PBT/ABS blend, and the compatibilized PBT/ABS/SAN-g-epoxy compositions were prepared by reactive extrusion in a Coperion Werner & Pfleiderer (Stuttgart, Germany) co-rotating twin-screw extruder, model ZSK, equipped with screws of 18 mm in diameter and a length-to-diameter ratio (L/D) of 40. The screw configuration was designed with mixing elements that promote dispersive and distributive actions, favoring the homogenization of the polymeric phases. The processing was carried out with a feed rate of 3 kg/h and a screw speed of 250 rpm. The temperature profile along the barrel zones was set to 210, 220, 230, 230, 240, 250, and 250 °C. After extrusion, the extruded strands were cooled in a water bath, subsequently dried using compressed air flow, and then pelletized in order to obtain granules suitable for the subsequent injection molding stage.

### 4.3. Injection Molding

Before the injection molding stage, the pellets obtained after extrusion were again subjected to drying in an oven at 80 °C under vacuum for 24 h. The specimens were produced using an Arburg (Loßburg, Germany) injection molding machine, model Allrounder 207C Golden Edition. The molding process was carried out with the barrel temperature profile set to 240, 240, 250, 250, and 250 °C. During molding, an injection pressure of 1300 bar and a holding pressure of 1000 bar were applied. The mold temperature was maintained at 50 °C, with a cooling time of 30 s and an injection speed of 10 m/min. The obtained specimens were subsequently used for characterization tests, conducted according to the corresponding technical standards.

### 4.4. Sample Characterization

The investigation of the in situ reactivity of the SAN-g-Epoxy compatibilizer in the PBT/ABS system was conducted through tests in a Haake torque rheometer (Polylab QC, Thermo Scientific, Waltham, MA, USA). The measurements were performed at 250 °C, with a rotational speed of 60 rpm, for a total processing time of 10 min. The test began after the mixing chamber, attached to the internal mixer and equipped with roller-type rotors, reached a temperature of 250 °C.

The rheological characterization under dynamic oscillatory conditions was carried out using an Anton Paar MCR 702 rheometer (Anton Paar GmbH, Graz, Austria), employing a parallel-plate geometry with a diameter of 25 mm and a plate gap of 1 mm. The tests were performed at 250 °C, with an angular frequency sweep ranging from 0.1 to 600 rad/s. The applied strain was 5%, previously established within the linear viscoelastic region of the material. For the analysis, samples obtained by machining from specimens originally prepared for the impact test were used.

The identification of chemical groups was performed by FTIR-ATR (Attenuated Total Reflectance) using a Bruker Alpha II spectrometer (Bruker Optik GmbH & Co. KG, Leipzig, Germany). The spectra were obtained in the range of 4000–400 cm^−1^, with a resolution of 4 cm^−1^ and 32 scans per sample, in order to improve the signal-to-noise ratio.

Izod impact strength was determined according to ASTM D256 using a CEAST RESIL 5.5 (CEAST S.p.A., Pianezza, Italy) impact tester equipped with a 2.75 J pendulum at room temperature. Eight specimens were tested for each formulation, and the results were reported as the average of the obtained values.

The tensile properties were determined according to ASTM D638 using a universal testing machine (Oswaldo Filizola, São Paulo, Brazil, model BME), with a crosshead speed of 50 mm/min and a 20 kN load cell. The results correspond to the average of eight specimens tested at room temperature.

Dynamic mechanical thermal analysis (DMTA) was performed using a TA Instruments rheometer (TA Instruments, New Castle, DE, USA), model HR-30, operating in dual cantilever bending mode. During the test, the samples were subjected to programmed heating from 30 to 120 °C, at a heating rate of 5 °C/min. The measurements were carried out at a frequency of 1 Hz under an air atmosphere. The test was performed with two samples to evaluate reproducibility and report the parameters in Table 1.

Thermomechanical resistance was evaluated by the heat deflection temperature (HDT), according to ASTM D648, using a CEAST HDT 6 VICAT (CEAST S.p.A., Pianezza, Italy) apparatus. The test was performed under a stress of 1.82 MPa with a heating rate of 120 °C/h, recording the temperature at which the deflection reached 0.25 mm in a silicone oil bath.

Thermal characterization was performed by DSC (Shimadzu DSC-60 Plus, Kyoto, Japan) under a N_2_ atmosphere (50 mL/min). The program included heating, cooling, and a second heating cycle between 30 and 250 °C at a rate of 10 °C/min, with a 2 min isothermal hold. Approximately 3 mg of sample was used, and the degree of crystallinity (Xc) was calculated from the melting enthalpy according to Equation (1):

(1) $$X_{c}\;=\;\frac{{∆H}_{m}}{w\times {∆H}_{100\%}}\times 100\%$$

In Equation (1), ΔH_m_ corresponds to the melting enthalpy obtained in the second heating cycle, w represents the mass fraction of PBT in the formulation, and ΔH_100_% corresponds to the melting enthalpy of 100% crystalline PBT (140 J/g) [86].

The morphological characterization of the fracture surfaces generated during the impact test was performed by field emission scanning electron microscopy (FEG-SEM) using a TESCAN VEGA 4 microscope (TESCAN, Kohoutovice, Czech Republic). The analyses were conducted under high vacuum conditions with an accelerating voltage of 5 kV. Prior to observation, the samples were coated with a thin gold layer with an approximate thickness between 20 and 30 nm in order to provide higher electrical conductivity and improve image definition. The selective extraction of the ABS phase was carried out using N-methyl-2-pyrrolidone (NMP) as the solvent. Initially, the fracture surfaces of the samples, obtained after the impact test, were continuously immersed in NMP at room temperature for a period of 5 days. To maximize the efficiency of extracting the dispersed ABS phase, the solvent was renewed daily, promoting the progressive dissolution of the component. After the immersion period, the samples were removed from the solvent and dried in a forced-air circulation oven at 50 °C for 24 h. Subsequently, morphological analysis was performed to evaluate the distribution and size of the dispersed phase. The average size of the ABS phase was determined using the image analysis software associated with the FE-SEM equipment, which provides greater accuracy and reliability in measuring particle dimensions.

## 5. Conclusions

This study demonstrated that the incorporation of 5 phr of the SAN-g-Epoxy compatibilizer was an effective strategy to promote interfacial compatibilization in PBT/ABS blends, resulting in significant improvements in mechanical and thermomechanical properties, as well as morphological stability. Torque rheometry analyses showed an increase in torque after the addition of SAN-g-Epoxy, indicating the occurrence of interactions between the components. This behavior was corroborated by FTIR results, which revealed an intensification of the carbonyl band associated with the possible formation of ester bonds during reactive processing. Compatibilization directly influenced the modification of the morphology of the polymer blends. SEM micrographs showed a significant refinement of the dispersed ABS phase, with the average particle size decreasing from approximately 4.34 µm in the non-compatibilized blend to around 0.47–0.54 µm in the compatibilized systems. This reduction in ABS inclusion size enhanced the interfacial area and morphological stabilization of the compatibilized blends, contributing to improved stress transfer between phases and energy dissipation mechanisms during fracture. As a result, impact strength increased significantly, from 63.4 J/m for neat PBT to values reaching up to 206.7 J/m, depending on the mixing sequence. Simultaneously, tensile properties were preserved or even enhanced, with elastic modulus values comparable to or higher than neat PBT and maintenance of tensile strength. In addition, significant gains in thermomechanical resistance, as measured by HDT, were observed for all produced blends. This behavior demonstrates that the compatibilization strategy allowed for achieving a balance between stiffness, strength, HDT, and toughness, an essential characteristic for the development of engineering polymeric materials.

Among the different mixing routes, the simultaneous PBT/ABS/SAN-g-Epoxy sequence exhibited the best overall balance of properties, suggesting it as the most suitable approach for structural and engineering applications requiring high mechanical and thermomechanical performance. Furthermore, this route requires only a single processing step in the extruder, contributing to reduced energy consumption and operational costs. Overall, the reactive compatibilization strategy allowed for achieving an optimal combination of stiffness, strength, toughness, and thermal stability, highlighting its potential for the development of engineering polymeric materials.

## Figures and Tables

**Table 1:** Storage modulus and glass transition temperatures obtained by DMTA.

Samples	E′ (MPa) (30 °C)	T_g_^PBT^ (°C)	T_g_^ABS^ (°C)
PBT	1156.7 ± 23.1	63.2 ± 0.2	-
PBT/ABS	883.5 ± 42.4	63.1 ± 0.3	113.0 ± 1.1
PBT/ABS/SAN-g-Epoxy	1254.1 ± 13.7	62.7 ± 0.1	112.9 ± 1.2
(PBT/ABS) + SAN-g-Epoxy	1189.2 ± 20.2	62.6 ± 0.2	112.8 ± 1.4
(PBT/SAN-g-Epoxy) + ABS	1143.6 ± 8.1	61.2 ± 0.1	113.1 ± 0.8
(ABS/SAN-g-Epoxy) + PBT	1172.3 ± 15.2	61.9 ± 0.2	112.8 ± 1.1

**Table 2:** Thermal properties by DSC for PBT and the polymer blends.

Samples	T_c_ (°C)	T_m_ (°C)		X_c_ (%)
		T_m_^1^ (°C)	T_m_^2^ (°C)	
PBT	194.0	215.0	223.8	22.7
PBT/ABS	193.7	213.9	223.5	29.9
PBT/ABS/SAN-g-Epoxy	197.1	216.3	223.3	22.1
(PBT/ABS) + SAN-g-Epoxy	197.1	216.3	223.2	22.0
(PBT/SAN-g-Epoxy) + ABS	197.9	217.0	222.8	27.6
(ABS/SAN-g-Epoxy) + PBT	197.6	216.8	223.5	26.9

## Data Availability

The original contributions presented in this study are included in the article. Further inquiries can be directed to the corresponding author.
